# Expanding Access to a New, More Affordable Levonorgestrel Intrauterine System in Kenya: Service Delivery Costs Compared With Other Contraceptive Methods and Perspectives of Key Opinion Leaders

**DOI:** 10.9745/GHSP-D-15-00327

**Published:** 2016-08-11

**Authors:** Kate H Rademacher, Marsden Solomon, Tracey Brett, John H Bratt, Claire Pascual, Jesse Njunguru, Markus J Steiner

**Affiliations:** a FHI 360, Raleigh-Durham, NC, USA; b Marie Stopes International, London, United Kingdom; c Marie Stopes Kenya, Nairobi, Kenya

## Abstract

**Background:**

The levonorgestrel intrauterine system (LNG IUS) is one of the most effective forms of contraception and offers important non-contraceptive health benefits. However, it is not widely available in developing countries, largely due to the high price of existing products. Medicines360 plans to introduce its new, more affordable LNG IUS in Kenya. The public‐sector transfer price will vary by volume between US$12 to US$16 per unit; for an order of 100,000 units, the public-sector transfer price will be approximately US$15 per unit.

**Methods:**

We calculated the direct service delivery cost per couple-years of protection (CYP) of various family planning methods. The model includes the costs of contraceptive commodities, consumable supplies, instruments per client visit, and direct labor for counseling, insertion, removal, and resupply, if required. The model does not include costs of demand creation or training. We conducted interviews with key opinion leaders in Kenya to identify considerations for scale-up of a new LNG IUS, including strategies to overcome barriers that have contributed to low uptake of the copper intrauterine device.

**Results:**

The direct service delivery cost of Medicines360’s LNG IUS per CYP compares favorably with other contraceptive methods commonly procured for public-sector distribution in Kenya. The cost is slightly lower than that of the 3-month contraceptive injectable, which is currently the most popular method in Kenya. Almost all key opinion leaders agreed that introducing a more affordable LNG IUS could increase demand and uptake of the method. They thought that women seeking the product’s non-contraceptive health benefits would be a key market segment, and most agreed that the reduced menstrual bleeding associated with the method would likely be viewed as an advantage. The key opinion leaders indicated that myths and misconceptions among providers and clients about IUDs must be addressed, and that demand creation and provider training should be prioritized.

**Conclusion:**

Introducing a new, more affordable LNG IUS product could help expand choice for women in Kenya and increase use of long-acting reversible contraception. Further evaluation is needed to identify the full costs required for introduction—including the cost of demand creation—as well as research among potential and actual LNG IUS users, their partners, and health care providers to help inform scale-up of the method.

## Abstract (precis)

At a public-sector transfer price of US$15 per unit, the direct service delivery cost of Medicines360's levonorgestrel intrauterine system (LNG IUS) per couple-years of protection is comparable with the cost of other contraceptive products commonly procured in Kenya. Interviews with key opinion leaders suggest that introduction of a more affordable LNG IUS could help increase demand for the method.

## Teaser

At a public-sector transfer price of US$15 per unit, the direct service delivery cost of Medicines360's levonorgestrel intrauterine system (LNG IUS) per couple-years of protection would be comparable with the cost of other contraceptive products commonly procured in Kenya. Interviews with key opinion leaders suggest that introduction of a more affordable LNG IUS could help increase demand for the method.

## INTRODUCTION

The levonorgestrel intrauterine system (LNG IUS) is one of the most effective forms of reversible contraception^1^^,^^2^ and is increasingly popular among women worldwide.^3^ The product offers a number of advantages beyond its high effectiveness, including reduction of menstrual blood loss and cramps, fewer side effects compared with some other hormonal methods, and possible alleviation of anemia in some populations—all of which could provide substantial benefits to women in developing countries.^4^^,^^5^ However, the high price of Mirena—an LNG IUS labeled for 5 years of use that is manufactured by Bayer HealthCare Pharmaceuticals and has been commercially available since 1990—has meant that its availability in developing countries has been extremely limited.^3^^,^^6^^,^^7^

Medicines360, a global nonprofit pharmaceutical company based in the United States, is poised to introduce a new, more affordable, highly effective LNG IUS in low-resource settings. Medicines360's mission is to expand access to medicines regardless of socioeconomic status, insurance coverage, or geographic location. In February 2015, Medicines360 and their partner Allergan (formerly Actavis) received U.S. Food and Drug Administration (FDA) approval for their LNG IUS, marketed as LILETTA in the United States, to prevent pregnancy for up to 3 years. Clinical trials are ongoing that will evaluate effectiveness for up to 7 years, with the expectation that the labeled duration of use will be extended.^8^ The product will be sold under the trade name Avibela in developing country markets. It is a T-shaped, intrauterine system loaded with 52 mg of levonorgestrel and is designed to provide a steady, localized release of approximately 20 mcg of LNG per day, which is comparable to Mirena. The product has already been approved in approximately 20 countries, including in the United Kingdom where it is approved for treatment of menorrhagia and 3 years of contraception under the brand name Levosert.^9^^,^^10^ In 2014, with funding from the Reproductive Health Supplies Coalition and the Bill & Melinda Gates Foundation, a new partnership was launched between FHI 360 and Marie Stopes International (MSI)/Marie Stopes Kenya (MSK) to develop an introduction strategy in Kenya for Medicines360’s new LNG IUS.^11^^,^^12^

In 2015, the U.S. FDA approved Medicine360’s LNG IUS to prevent pregnancy for up to 3 years. Ongoing clinical trials are evaluating effectiveness for up to 7 years.

### Current Landscape in Kenya

The modern contraceptive prevalence rate (mCPR) among married women of reproductive age in Kenya was 39.4% in 2008–2009 and rose to 53.2% by 2014, according to Demographic and Health Survey data.^13^^,^^14^ This increase is also reflected in findings from the most recent Performance Monitoring and Accountability 2020 (PMA2020) survey as of publication of this article, which reported an mCPR of 63.1% in Kenya among married women in 2015.^15^ However, copper intrauterine devices (IUDs) remain an underutilized method in Kenya, with 3.4% of married women of reproductive age using the method in 2014 and 4.8% in 2015.^14^^,^^15^ In contrast, use of implants has increased steadily over the last decade; prevalence increased from 1.9% in 2008–2009 to 9.9% in 2014 to 16.1% in 2015. Injectables continue to dominate the market, with 26.4% of married women of reproductive age using the method in 2014 and 29.2% in 2015 (Table 1).^13^^-^^15^

Copper IUDs are currently an underutilized method in Kenya.

**TABLE 1.** Contraceptive Prevalence Among Married Women of Reproductive Age in Kenya, 1988–2015

Percentage of Married Women Using:				
Survey Year	Any Modern Method	Injectable	Copper IUD	Implant
1988–1989	17.9	3.3	3.7	0.0
1993	27.3	7.2	4.2	0.0
1998	31.5	11.8	2.7	0.8
2003	31.5	14.3	2.4	1.7
2008–2009	39.4	21.6	1.6	1.9
2014	53.2	26.4	3.4	9.9
2015	63.1	29.2	4.8	16.1

Abbreviation: IUD, intrauterine device.

Source of data: 2015 survey from Performance Monitoring and Accountability 2020 (PMA2020)^15^; all other surveys from Demographic and Health Surveys.^16^

The Mirena is not currently offered in the public sector in Kenya and is only available on a limited basis in the private sector. According to an assessment of the current sales of Mirena conducted by MSK in 2014, its price ranges from US$56 to US$194 in private-sector clinics in Kenya depending on the location and type of the clinic (in some locations, clients could negotiate a discount ranging from US$6 to US$22).^17^ To date, limited quantities of free LNG IUS commodities have been donated through the International Contraceptive Access (ICA) Foundation for distribution in Kenya by NGOs. The ICA Foundation was established in 2003 and is a partnership between Bayer HealthCare and the Population Council. Approximately 70,000 units have been donated worldwide since 2005.^18^ Between 2008 and 2011, MSK provided approximately 5,000 donated units of the LNG IUS in Kenya.^19^ However, the ICA Foundation does not provide support for marketing or training which has limited the ability of NGOs to scale-up the method.^7^

A recent study of women using the ICA Foundation product in Kenya showed high acceptability and uptake of the method. Among 671 postpartum women offered a range of short-acting and long-acting methods at no charge, 16% chose the LNG IUS (N = 109) (all methods were offered to the clients at this facility for free).^6^ (Only a subset [n = 93], however, initiated use of the method, either due to medical contraindications or personal reasons.^20^) Approximately one-third of LNG IUS users in the study indicated that if the product had not been available, they would have chosen a shorter-acting method; only 21% said they would have used a copper IUD, indicating that the hormonal product could potentially fill a niche in the market that is not currently filled by the copper IUD.^6^ In a follow-up assessment of continuation rates, 89% of LNG IUS users (82 of 92 women; 1 woman was lost to follow-up) were still using the method after 1 year, which was comparable to the continuation rate of subdermal implants at 91% (179 of 196 women). Among the 79 LNG IUS users who provided information about their experiences with the product, 87% reported being “very satisfied” with the method at 12 months, with 13% “somewhat satisfied” (similar to 84% and 12% among implant users, respectively; uptake of copper IUDs was low and continuation rates were not reported).^20^

To determine if Medicines360’s LNG IUS would be cost-competitive compared with other contraceptive products if introduced in Kenya, we conducted an analysis of the direct service delivery costs of various family planning methods that are currently being offered or are likely to be introduced there soon. In addition, staff from FHI 360 and MSK conducted interviews with key opinion leaders, which explored current barriers to use of the copper IUD and key considerations for successful scale-up of a new hormonal product.

## METHODS

We revised and expanded an earlier analysis conducted by Tumlinson and colleagues of direct service delivery costs of contraceptives per couple-years of protection (CYP).^21^ We included Medicines360’s LNG IUS and updated cost inputs for Kenya. Medicines360 has exclusive distribution rights for the product in 61 countries in Africa and South Asia, and their public‐sector transfer price (the price from the supplier, Medicines360, to the distributor) in these countries will vary based on volumes between US$12 and US$16 per unit. The analysis conducted reflects a public‐sector transfer price of US$15 per unit based on a weighted average for an order of 100,000 units.^10^ (The distributor may then add additional margins when selling the product in-country, per the terms in the agreement with the supplier.)

FHI 360/Kenya and MSK staff also identified and interviewed key opinion leaders to better understand the potential demand for a more affordable LNG IUS in Kenya through the public and private sectors.

### Calculating Direct Service Delivery Costs

Elements of direct service delivery costs included commodity costs to international procurers as well as costs of consumable supplies, estimated costs of instruments per client visit, and costs of direct labor for counseling, insertion, removal, and resupply if required.

Consumable supplies include supplies such as sterile gloves, sharps boxes, syringes, scalpel blades, sterile drapes, sanitary pads, and analgesics, when relevant for the various contraceptive methods. Instruments included items such as forceps, bowls, scalpel handles, scissors, and specula.^22^ For instruments and consumable supplies that could be used for multiple procedures, we divided the total cost of the product by an estimated number of procedures to derive a unit cost. We did not include supplies that have a negligible unit cost (including antiseptic, soap, and iodine) in the model. Supplies for both insertion and removal, if relevant, were included. Costs of consumable supplies were taken from the United Nations Population Fund’s (UNFPA) AccessRH Product Catalogue^23^ and the IDA Foundation’s Electronic Price Indicator.^24^ Cost of instruments required for provision of long-acting and permanent methods was obtained through personal communication with an international medical instrument manufacturer (Shendu Pak).

Contraceptives currently available in the public sector in Kenya were included, as well as products such as the Sayana Press injectable that are likely to be introduced within the next several years. Costs for all contraceptive commodities came from UNFPA’s AccessRH Product Catalogue^23^ or from information in the public domain provided by the supplier in the case of Medicines360’s LNG IUS.^10^ For female and male condoms, we used an average price of the various condoms listed in the catalog. Table 2 includes a summary of the commodity costs and consumable supplies used in the model. Supply and commodity costs can fluctuate over time; the prices used in the model were accessed in September 2015.

**TABLE 2.** Per Unit Costs of Commodities, Consumable Supplies, and Instruments Included in the Model (US$)

Method	Commodity	Consumables for Insertion/Initial Provision	Consumables for Resupply	Consumables for Removal	Instruments
COCs	$0.27	NA	NA	NA	NA
Copper IUD	$0.35	$0.90	NA	$0.90	$0.13
DMPA injectable	$0.72	$0.34	$0.34	NA	NA
Female condom	$0.45	NA	NA	NA	NA
Female sterilization	NA	$6.64	NA	NA	$0.15
Implanon implants	$8.50	$1.32	NA	$1.32	$0.03
Jadelle implants	$8.50	$1.32	NA	$1.32	$0.03
LNG IUS	$15.00	$0.90	NA	$0.90	$0.13
Male condom	$0.03	NA	NA	NA	NA
Male sterilization	NA	$4.67	NA	NA	$0.03
NET-EN injectable	$1.15	$0.34	$0.34	NA	NA
Sayana Press injectable	$1.00	$0.34	$0.34	NA	NA
Sino-implant (II) implants	$8.00	$1.32	NA	$1.32	$0.03

Abbreviations: COCs, combined oral contraceptives; DMPA, depot medroxyprogesterone acetate; IUD, intrauterine device; LNG IUS, levonorgestrel intrauterine system; NA, not applicable; NET-EN, norethisterone enanthate.

Source of data: UNFPA AccessRH Product Catalog,^23^ IDA Foundation E-catalogue,^24^ Stephens,^10^ and personal communication with international medical supplier Shendu Pak (for cost of instruments required for long-acting and permanent methods). Supply and commodity costs can fluctuate over time; the prices used in the model were accessed in September 2015.

We also included labor time for counseling, insertion, removal, and resupply (if applicable) in the model. Assumptions about labor times were taken from Futures Institute’s (now Avenir Health’s) OneHealth Tool.^25^ For example, the model assumes that IUD insertion would require 20 minutes for counseling and 15 minutes for insertion, as well as 1 follow-up visit for 10 minutes and then 10 minutes for removal. We applied the same labor times to the LNG IUS. For all methods except male and female sterilization and male condoms, we assumed that a nurse-midwife would provide the methods, which is allowed according to national policies and is common practice in the public and private sectors in Kenya.^26^ For both male and female sterilization, it was assumed that a physician would spend 30 minutes on the procedure and a nurse-midwife would provide 30 minutes of counseling (for the woman and her partner together), 30 minutes for support to a physician during the procedure, and 10 minutes for a follow-up appointment. For male condoms, we assumed that counseling and method provision would be completed by an unpaid, volunteer health worker; this includes 20 minutes for the first visit for counseling and provision and then follow-up visits of 5 minutes each. For female condoms, we assumed that counseling and provision would be offered by a nurse-midwife. We obtained 2015 salaries for Kenya based public-sector staff through personal correspondence with staff at FHI 360/Kenya. The model reflects a monthly salary of 51,590 KES (US$552) for a nurse-midwife, and 75,840 KES (US$811) for a physician. (These salaries include standard allowances provided to employees in the Kenyan public health system, such as uniform or leave allowance). Table 3 summarizes the total time required by cadre of provider.

**TABLE 3.** Total Labor Time Included in the Model for Counseling, Insertion, Removal, and/or Resupply by Method and Provider Cadre

	Nurse-Midwife			Physician	Unpaid Health Worker	
Method	Counseling and Method Provision	Follow-up or Resupply	Removal	Method Provision	Counseling and Method Provision	Resupply
COCs	25 min	5 min	NA	NA	NA	NA
DMPA injectable	25 min	5 min	NA	NA	NA	NA
Female condom	20 min	5 min	NA	NA	NA	NA
Female sterilization	60 min	10 min	NA	30 min	NA	NA
Implanon implants	35 min	10 min	15 min	NA	NA	NA
Copper IUD	35 min	10 min	10 min	NA	NA	NA
Jadelle implants	35 min	10 min	15 min	NA	NA	NA
LNG IUS	35 min	10 min	10 min	NA	NA	NA
Male condom	NA	NA	NA	NA	20 min	5 min
Male sterilization	60 min	10 min	NA	30 min	NA	NA
NET-EN injectable	25 min	5 min	NA	NA	NA	NA
Sayana Press injectable	25 min	5 min	NA	NA	NA	NA
Sino-implant (II) implants	35 min	10 min	15 min	NA	NA	NA

Abbreviations: COCs, combined oral contraceptives; DMPA, depot medroxyprogesterone acetate; IUD, intrauterine device; LNG IUS, levonorgestrel intrauterine system; NA, not applicable; NET--EN, norethisterone enanthate.

Source of data: Futures Institute.^25^

We used standard CYP conversion factors for each method.^27^ For short‐ and mid-acting methods, we aggregated costs of visits made throughout the year to achieve 1 CYP. For long-acting methods, we divided the costs by the appropriate conversion factor. We used a conversion factor of 3.3 years for the LNG IUS, which assumes it will be registered as a 5-year product in Kenya. The model does not reflect other costs required to support product introduction including costs for demand creation and provider training.

### Interviews With Key Opinion Leaders

Staff from FHI 360 and MSK conducted 13 semi-structured interviews with key opinion leaders from a variety of organizations, including the Ministry of Health (n = 2), NGOs (n = 4), international donors and normative bodies such as United Nations bodies (n = 4), a private-sector franchisee (n = 1), a pharmaceutical distributor (n = 1), and a member of a national reproductive health society (n = 1). The key opinion leaders were identified and purposively selected based on their knowledge and expertise within the reproductive health field. The interviewers used a standard interview guide with open-ended questions. Interviewers asked respondents about their perceptions of the current and future landscape for IUDs, potential demand for a new LNG IUS, and potential users’ needs and preferences. All interviews were conducted in English and were audio-recorded and transcribed. Staff based in the United States and United Kingdom categorized the responses in Microsoft Excel and selected illustrative quotations that reflected common themes.

## RESULTS

The Figure shows that the direct service delivery cost of Medicines360’s LNG IUS per CYP would compare favorably with costs of other contraceptive methods commonly procured for public-sector distribution in Kenya. Assuming Medicines360’s LNG IUS will ultimately be registered as a 5-year product, its direct service delivery cost per CYP (US$6.34) would be slightly lower than that of the 3-month contraceptive injectable, depot medroxyprogesterone acetate (DMPA) (US$7.07), which is currently the most popular method in Kenya, making up 46.2% of the method mix among married women of reproductive age in 2015.^15^

Direct service delivery cost per CYP of Medicines360’s LNG IUS would compare favorably with costs of other contraceptive methods distributed in Kenya.

**FIGURE.**
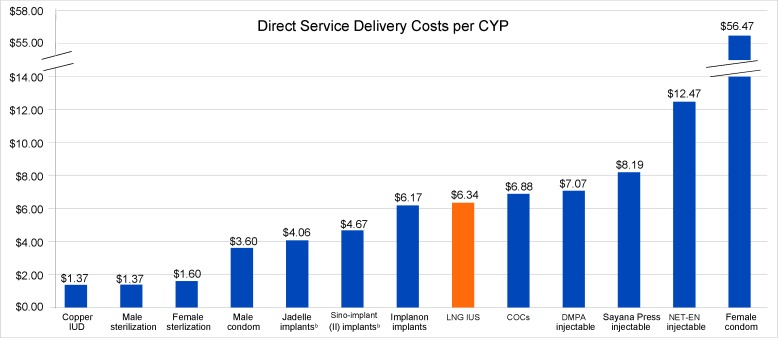
Direct Service Delivery Cost of the LNG IUS per CYP^a^ Compared With Cost per CYP of Other Contraceptive Methods (2015 US$) Abbreviations: COCs, combined oral contraceptives; CYP, couple-year of protection; DMPA, depot medroxyprogesterone acetate; IUD, intrauterine device; LNG IUS, levonorgestrel intrauterine system; NET‐EN, norethisterone enanthate. ^a^ The public‐sector transfer price for Medicines360’s LNG IUS depends on volumes. The calculated LNG IUS cost per CYP of US$6.34 in this chart reflects a US$15 per unit public‐sector transfer price based on a weighted average for an order of 100,000 units. ^b^ Although the commodity cost of Sino-implant (II) implants (US$8 per unit) is lower than the commodity cost of Jadelle implants (US$8.50 per unit), Sino-implant (II) is labeled for 4 years of use with a CYP conversion factor of 3.2 years while Jadelle is labeled for 5 years of use with a CYP conversion factor of 3.8 years. Thus, the cost per CYP for Jadelle is slightly lower than that of Sino--implant (II).

At the time of this publication, Medicines360’s product was registered for 3 years of use in the United States and the United Kingdom. However, as noted, clinical trials in the United States are ongoing with the expectation that the labeled duration of use will be extended up to 7 years. The Medicines360 LNG IUS contains 52 mg of levonorgestrel, which is the same amount of active pharmaceutical ingredient as the Mirena, which is registered for 5 years of use. The timeline for product registration in Kenya is uncertain, and it is unknown if Medicines360’s LNG IUS will be initially registered there as a 3-year or 5-year product. If it is initially registered as a 3-year product and the CYP conversion factor for Implanon is applied in the analysis (i.e., a conversion factor of 2.5 years), then the cost per CYP of Medicines360’s LNG IUS would be US$8.37 (not shown in the Figure).

### Perspectives of Key Opinion Leaders: Current Barriers to Uptake of the Copper IUD

All of the key opinion leaders were in agreement that there is potential to increase overall use of IUDs in Kenya. They noted that Kenyan women increasingly want to space and limit their children and are also demanding more choice of contraceptive methods, particularly long-acting methods. (According to the 2015 surveys from PMA2020, 15.9% of married women have an unmet need for family planning, with 7.2% having an unmet need for limiting and 8.7% for spacing. Among married women, 20.2% are currently using long-acting methods.^15^) However, respondents also noted that IUD usage has remained relatively low in contrast to other methods, including implants, which have been a focus of government advocacy efforts in recent years. All of the key opinion leaders attributed the low usage of IUDs to a general lack of knowledge and enduring myths and misconceptions about IUDs among women, providers, and the general public. Respondents reported common myths among women, such as the belief that the IUD can get lost in the body or become implanted in an infant during birth, as well as concerns about impact on sexual pleasure, including concerns that sexual partners can feel the strings. Key opinion leaders reported that providers often erroneously think that the IUD is only appropriate for multiparous women, and are confused about the correct timing of insertion.

Insufficient training for health care providers was also commonly seen as a barrier to IUD scale-up, and key opinion leaders noted that insertion skills and confidence are often lacking. Additional challenges mentioned included limited availability of IUD commodities, a lack of insertion equipment, insufficient space within clinics to offer IUDs, and the longer time required to insert IUDs compared with shorter-acting methods such as injectables. Several respondents also cited the increased bleeding that is often a side effect of copper IUDs as a reason they have not become more popular with women.

### Current Perceptions of and Experiences With the Mirena

Almost all of the key opinion leaders had heard of Mirena and had at least a basic understanding of the product (e.g., all were aware that it is a hormonal IUD). The majority of respondents reported that the product is currently used by women of middle and high socioeconomic status, and several mentioned that users are typically employed and live in urban areas. Key opinion leaders noted that key barriers to uptake of the LNG IUS have been both the lack of availability of the product in the public sector and the high prices of the commodity and insertion fees. For example, one respondent stated:

[A barrier which] is extremely important is the cost … I can talk even now as a private practitioner. You have a costing that goes anything from 6,000 to 12,000 KES [US$64–US$128]. That’s the range, that’s the whole procedure; it’s all loaded to the patient. I have an idea of the range of the market because the prices also fluctuate depending on where you are sourcing the product from. So that’s a big, big, big barrier. And I must say, it’s more expensive in the bigger the hospital wherever you’re going to put it in. So that’s one of the biggest limitation to its use, cost. It’s not in the public domain.

When asked how Mirena has been perceived by providers and clients who know about it, the most common response was that the product is known for offering non-contraceptive benefits, and several specifically mentioned reduced bleeding.

### Potential Demand for a More Affordable LNG IUS in the Public and Private Sectors

Almost all of the respondents agreed that introducing a more affordable LNG IUS could increase uptake of the method. They also emphasized the importance of addressing the barriers that have historically contributed to low uptake of IUDs in order to ensure successful introduction and scale-up of a new product. For example, one respondent said:

I think once we’ve cleared … the myths and misconceptions about IUCDs [intrauterine contraceptive devices] in general and we applaud the benefits … demand is there. All people need to be assured that you don’t have to swallow anything, you don’t have any systemic side effects. I think that’s what people are concerned about: weight gain, bleeding, and those kinds of things. Then I think we can work on the cost, and once we address the cost side of things, [and] when people have information, then we expect the demand will be high.

Key opinion leaders emphasized the importance of addressing the barriers that have contributed to low uptake of IUDs.

One respondent expressed skepticism that introducing a more affordable LNG IUS would be sufficient to increase uptake of the method, emphasizing that the LNG IUS would still face service delivery challenges similar to the copper IUD. This individual said:

Issues around [copper] IUDs and the LNG IUS would be very similar. [The copper] IUD is generally good for the private sector and available free from the Health Ministry and the uptake is poor … So, I think it’s not just the price.

In general, the key opinion leaders agreed that an introduction strategy should include both the public and private sectors, as exemplified by this comment from one respondent:

Let’s go for the total market. We want this hormonal IUCD to [be made] available to the public and the private … sectors. Yes, let it be available.

There were differing opinions regarding whether uptake would be greatest in the public or private sector. Some felt that if the price were substantially reduced and the product were made available, the greatest opportunity to increase access would be through public-sector distribution, which would increase uptake among low-income women. Others felt that the greatest opportunity lies within the private sector, and several respondents indicated that users would likely continue to be mid- to high-income women. Other key market segments identified included women seeking non-contraceptive health benefits, as well as postpartum women and adolescents.

The majority of key opinion leaders expressed the opinion that reduced bleeding would be viewed as a key advantage among potential users. Almost all respondents indicated that women would welcome reduced menstrual bleeding for lifestyle reasons such as increased convenience. Groups who would potentially find reduced bleeding attractive include students, career women who travel frequently, and female sex workers, as well as those who would benefit clinically, such as women suffering from heavy menses and those at risk of anemia. One respondent explained:

I’m looking at a woman in the village. You already have nutritional setbacks; you have the monthly menstruation that is an excessive amount. So [amenorrhea] is a plus, plus, plus! You’re reducing the anemia risk and the quality of life with pain.… [It] benefits business women and school girls … when you look at the cost-benefit there is a lot more to gain and with some degree of independence and freedom.

The majority of key opinion leaders thought that reduced bleeding would be perceived as an advantage among potential users.

Other product characteristics identified that would be attractive to women include reduced cramping, as well as its long-term duration of use and effectiveness.

### Key Considerations for Product Launch

Key opinion leaders were asked about the essential steps required to ensure the successful introduction of a new LNG IUS. They all agreed that effective demand creation is crucial; the need for education and awareness was mentioned as a priority by all respondents. For example, one respondent said:

[We need] to work on the myths and misconceptions … the communications aspect.

Key opinion leaders also emphasized the importance of working with a broad coalition of stakeholders to generate buy-in and support for introduction and scale-up of the method. For example, one key opinion leader said:

Start with the policy opinion leaders. Let them … secure buy-in from opinion leaders. What I mean is the MOH [Ministry of Health], the donors who support family planning, the key service providers of family planning … everybody.

Key opinion leaders also emphasized the importance of expanding and improving provider training. One respondent said:

For launching, one of the first steps is creating awareness, improving the capacity building through training. Training of providers is critical.

Other recommended strategies included ensuring that the product is available in the public sector as well as the private sector, that there are adequate supplies and equipment, that the LNG IUS is adequately reflected in national policies and guidelines, and that the product becomes prequalified by the World Health Organization. Several key opinion leaders acknowledged that challenges to introduction could include resistance from private-sector providers to a lower-cost LNG IUS because of the high margins they are currently able to charge.

## DISCUSSION

The direct service delivery costs per CYP of Medicines360’s LNG IUS will be competitive with other hormonal family planning commodities commonly procured by international donors and the government for public-sector distribution in Kenya. In addition to the very high effectiveness of the LNG IUS at preventing pregnancy, the LNG IUS’s non-contraceptive health benefits may make it an attractive investment for the government and donors. Realistically, the price of LNG IUS products including Medicines360’s will never be equivalent to that of the copper IUD—which can be procured for US$0.35.^23^ However, because the LNG IUS has a different side effect profile from the copper IUD (e.g., the hormonal product can lead to reduced menstrual bleeding, whereas the copper product is associated with heavier, prolonged menses),^3^ it is appropriate for decision makers to compare the direct costs of the LNG IUS with the full range of available family planning methods, including contraceptive implants and injectables, as well as to products that are likely to be introduced in Kenya soon, such as Sayana Press.

When asked which women would be most likely to use a new LNG IUS in Kenya, the key opinion leaders most frequently mentioned women who would be attracted to its non-contraceptive clinical benefits. Although Medicines360’s LNG IUS has a higher up-front commodity cost than the other contraceptive methods included in the analysis, the method could be positioned in Kenya as a “dual purpose” technology—that is, as a contraceptive product that can treat menorrhagia and has the potential to alleviate anemia. By way of comparison, female condoms—which are promoted as an important “multipurpose technology” that offer protection from both unintended pregnancy and transmission of HIV and other sexually transmitted infections—have a high direct cost per CYP of US$56, as illustrated in the Figure. Additional cost–benefit analyses of the LNG IUS conducted in Kenya and other developing countries are needed to compare the use of the product with other therapeutic interventions, including for treatment of heavy menstrual bleeding.^28^^-^^30^

The LNG IUS could be positioned in Kenya as a “dual purpose” technology.

In addition, an important finding from the qualitative interviews was the perception among the key opinion leaders that women would welcome reduced menstrual bleeding for lifestyle reasons. There is a lingering perception among some in the public health community that women in developing countries are unlikely to find contraception-induced amenorrhea acceptable. The responses from the key opinion leaders in Kenya challenge this assumption and instead suggest that some women in Kenya may seek out a method that is associated with a reduction in menstrual blood loss and cramping.

That said, because of the limited availability of the LNG IUS to date in most developing countries, including Kenya, it is unknown what true demand would be if the product were offered at scale. The uptake of the copper IUD has been low compared with other contraceptives, so it remains a question whether wide-scale introduction of a new LNG IUS would lead to increased use in Kenya. It is notable that almost all of the key opinion leaders agreed that the introduction of a more affordable LNG IUS would increase demand and uptake of the method. This perception is consistent with the findings of Hubacher and colleagues in an earlier study in Kenya, which showed that 16% of women selected the LNG IUS when it was offered as part of a broader method mix.^6^ At the same time, the key opinion leaders all cautioned that factors on both the demand and supply sides that have contributed to low uptake of copper IUDs in Kenya must be addressed for introduction of a new LNG IUS to be successful at scale.

### Limitations

Limitations of this assessment include that a small number of key opinion leaders were interviewed; their perspectives may not be representative of other stakeholders’ perspectives or adequately predict how clients and providers will react to the product. Interviews with women were not included in this analysis. Both quantitative and qualitative research is needed among current and potential LNG IUS users and their partners, as well as among health care providers. Most importantly, once the product begins to be introduced, monitoring uptake and evaluating acceptability will be critical both to inform additional scale-up in Kenya and to offer evidence and lessons learned for other countries in the region.

An additional limitation is that the analysis of the costs per CYP of various family planning methods includes only direct, variable costs for service delivery. We did not include fixed facility costs or costs for up-front and ongoing training and demand creation. The full costs of product introduction—including provision in various service delivery settings—will be important to document.

## CONCLUSIONS

Results from this early assessment indicate that introduction of a more affordable LNG IUS has the potential to increase access and choice for women in Kenya, and that the method’s side effect profile and the associated non-contraceptive health benefits may be attractive to women**.** As a follow-on to the costing assessment and interviews with key opinion leaders, an introduction strategy for Medicines360’s product in Kenya is being developed by MSI/MSK and FHI 360 with input from stakeholders. One goal of this exercise is to identify the steps needed to address the potential barriers to scale-up identified by the key opinion leaders. These include ensuring adequate provider training, addressing myths and misconceptions among both clients and providers, and increasing awareness of the product among policymakers, the health care community, and the general public.

## Notes

**Peer Reviewed**
